# Naringenin restores osteogenic differentiation in TNF-α-Treated bone marrow mesenchymal stem cells by targeting AKR1B1

**DOI:** 10.1038/s41598-025-22035-w

**Published:** 2025-10-31

**Authors:** Bin He, Feng He, Huimin Li, Leyi Huang

**Affiliations:** Department of Orthopaedic Surgery, The Fourth Affiliated Hospital of School of Medicine, International School of Medicine, International Institutes of Medicine, Zhejiang University, Yiwu, 32200 China

**Keywords:** Naringenin, AKR1B1, Osteogenic differentiation, TNF-α, hBMSCs, Oxidative stress, Mesenchymal stem cells, Stem-cell differentiation

## Abstract

In chronic inflammatory microenvironments, TNF-α disrupts bone remodeling by suppressing osteogenic differentiation in human bone marrow mesenchymal stem cells (hBMSCs), while conventional TNF-α inhibitors lack osteoprotective effects. This study reveals that naringenin counteracts TNF-α-induced osteogenic suppression by potentially targeting AKR1B1 to restore redox balance and inhibit NF-κB-mediated inflammation. In vitro experiments demonstrated that naringenin restored osteogenic capacity in TNF-α-treated hBMSCs, enhancing ALP activity, mineralization, and expression of RUNX2/OCN while reducing IL-6/IL-1β levels. Mechanistically, naringenin scavenged free radicals, elevated SOD/CAT activity, and attenuated TNF-α-driven ROS accumulation. Bioinformatics analysis identified TNF-α-activated NF-κB signaling and upregulation of the oxidative stress enzyme AKR1B1, with molecular docking confirming strong binding between naringenin and AKR1B1. This study pioneers AKR1B1 as a novel therapeutic target for inflammatory bone loss, demonstrating naringenin’s synergistic “antioxidant-anti-inflammatory-pro-osteogenic” effects. These findings provide a theoretical foundation for phytochemical applications in orthopedic therapies and highlight potential clinical translation strategies.

## Introduction

Chronic inflammatory microenvironments are pivotal drivers of impaired bone metabolism, contributing to clinically significant challenges such as osteoporosis, delayed fracture healing, and inflammatory osteolysis in conditions like rheumatoid arthritis and diabetic bone disease^1^. Pro-inflammatory cytokines disrupt the balance between osteogenesis and osteoclastogenesis, favoring bone resorption while suppressing osteoblast differentiation. Among these cytokines, tumor necrosis factor-alpha (TNF-α) plays a central role in bone remodeling. Elevated TNF-α levels inhibit osteogenic differentiation of bone marrow mesenchymal stem cells (BMSCs) by downregulating key transcription factors and activating NF-κB signaling, which amplifies inflammatory cascades and promotes osteoclastogenesis^2^. Clinical evidence further links TNF-α overexpression to poor bone repair outcomes, underscoring its therapeutic relevance^3^. While TNF-α inhibitors (e.g., biologics) mitigate inflammation, their immunosuppressive risks and limited capacity to directly restore osteogenesis necessitate alternative strategies targeting the molecular crosstalk between inflammation and osteoblast dysfunction.

It is noteworthy that the effect of TNF-α on osteogenic differentiation is not uniform and exhibits concentration-dependent characteristics^4^. Most studies indicate that high concentrations of TNF-α (typically > 10 ng/mL) strongly inhibit osteogenesis-related gene expression and induce apoptosis through sustained activation of inflammatory pathways such as NF-κB, thereby impairing bone formation^5^. For example, 10 ng/ml TNF-α inhibits the osteogenic differentiation ability of human periodontal stem cells by reducing the expression of miR-21, leading to an increase in the level of recombinant spry1, thereby inhibiting the production of ALP and RUNX2^6^. However, some studies have also reported that at lower concentrations (< 2 ng/mL) or upon short-term stimulation, TNF-α may conversely promote the proliferation and early differentiation of osteoprogenitor cells by activating essential intracellular signals, such as the MAPK pathway^7^. Research reports indicate that treating human bone marrow mesenchymal stem cells with 1 ng/ml TNF-α can activate the NF-κB pathway, inhibit the binding of peroxisome proliferator-activated receptor (PPAR)-γ and PPRE, and upregulate the mRNA levels of TNAP, COX-2, prostaglandin E2 synthase, and prostaglandin E2 synthase. Therefore, through this mechanism, the expression of TNAP increases in the osteoblast cell line MG-63, thereby enhancing cell mineralization^8^. This biphasic effect underscores the complex role of TNF-α in bone physiology and pathology. We hypothesize that transient inflammatory stimulation may facilitate the rapid initiation of bone repair. In contrast, chronic inflammation persistently alters the bone microenvironment, impairing the self-repair capacity of bone tissue and leading to progressively destructive outcomes.

From the perspective of physiological regulation, osteoblast differentiation is a precisely regulated cascade process. The core transcription factor RUNX2 acts as the “master regulator of osteogenesis,” initiating the differentiation of mesenchymal stem cells into the osteoblastic lineage and regulating the expression of early osteogenic markers^9,10^. Osterix (OSX), acting downstream of RUNX2, is essential for osteoblast maturation and mineralization, and together they coordinate the expression program of osteoblast-specific genes^9^. Furthermore, reactive oxygen species (ROS) also play an important yet dual role in osteogenic maturation: physiological levels of ROS can act as second messengers to promote the activation of signaling pathways related to osteogenic differentiation, whereas excessively high levels of ROS (e.g., under chronic inflammatory conditions) induce oxidative stress, impair mitochondrial function, and suppress the activity and expression of RUNX2 and OSX, ultimately leading to impaired osteogenic differentiation and reduced bone formation^11^. Therefore, identifying agents capable of simultaneously mitigating oxidative stress, suppressing inflammation, and promoting osteogenic differentiation is of critical importance for developing effective therapies against inflammation-associated bone loss.

Natural flavonoids with dual anti-inflammatory and osteoprotective properties have emerged as promising candidates to address this unmet need. Naringenin, a bioactive compound abundant in citrus fruits, exhibits potent antioxidative and anti-inflammatory effects, including scavenging reactive oxygen species, suppressing NLRP3 inflammasome activation, and inhibiting pro-inflammatory cytokine release^12^. Notably, recent studies suggest its regulatory effects on bone metabolism, such as enhancing osteoblast mineralization under physiological conditions^13–16^. At the molecular mechanism level, naringenin is known to exert osteoprotective effects by modulating multiple signaling pathways. For instance, it can activate the Wnt/β-catenin pathway to promote osteoblast differentiation and function, while simultaneously inhibiting NF-κB and MAPK inflammatory signaling pathways to alleviate inflammation-induced suppression of osteogenesis^17–19^. Moreover, studies have demonstrated that naringenin upregulates the expression of key osteogenic transcription factors RUNX2 and Osterix, and enhances alkaline phosphatase (ALP) activity and bone nodule mineralization^18^. Notably, naringenin also possesses the potential to modulate the balance of the RANKL/RANK/OPG system, thereby promoting osteogenesis while inhibiting excessive osteoclastic activity^20^. These multi-target characteristics make naringenin particularly suitable for intervening in TNF-α-mediated inflammatory bone loss. However, its precise molecular mechanisms in rescuing osteoblast function under inflammatory conditions remain unclear, particularly regarding direct interactions with TNF-α-mediated pathways. This knowledge gap limits the translation of naringenin’s potential into targeted therapies for inflammatory bone disorders.

Building upon this foundation, our study identifies naringenin as a potent modulator of TNF-α-induced osteogenic suppression, acting primarily through the targeting of AKR1B1. By integrating multi-omics data with functional experiments, we provide mechanistic insights into how naringenin rescues osteoblast differentiation in inflammatory microenvironments. These findings not only advance our understanding of flavonoid-based interventions for bone disorders but also highlight AKR1B1 as a novel therapeutic target for inflammatory bone diseases. This work bridges phytochemical research and clinical orthopedics, offering a foundation for developing naringenin as a multi-target agent to combat inflammation-driven bone loss.

## Materials and methods

### In vitro biocompatibility test

Human bone marrow-derived mesenchymal stem cells (hBMSCs) were derived from bone marrow flushes of clinical patients undergoing knee replacement. The study protocol was approved by the human research ethics committee of The Fourth Affiliated Hospital of Zhejiang University School of Medicine (approval number: K2024086) and the experimental process strictly followed the Declaration of Helsinki. Informed consent was obtained from all the partcipants and/or their legal guardian(s).

hBMSCs were maintained in α-MEM complete medium supplemented with 10% fetal bovine serum and 1% antibiotics. For the live/dead staining assay, cells were seeded into 24-well plates and divided into four groups: a control group, a group treated with 20 ng/mL TNF-α alone, a group receiving 20 ng/mL TNF-α with 20 µM naringenin (designated as “+”), and another group treated with 20 ng/mL TNF-α plus 50 µM naringenin (designated as “++”). The selection of 20 µM and 50 µM naringenin was based on concentrations previously established in studies examining the osteogenic effects of naringenin in human dental pulp stem cells (hDPSCs) and other relevant cell models^21,22^. After incubating for 48 h, the cells were incubated in the dark for 30 min with a staining solution containing 5 µM Calcein-AM and 0.6 µM propidium iodide (Life Technologies), following the manufacturer’s protocol. The resulting images of live and dead cells were then captured using an inverted microscope (LSM700, Zeiss, Germany).

For the CCK-8 assay, hBMSCs were first detached using trypsin and subsequently reseeded into 96-well plates. At time points of 24, 48, and 72 h, 10 µL of CCK-8 reagent was added to each well, and the plates were incubated at 37 °C. Finally, the absorbance was measured at 450 nm with a microplate reader, allowing for quantitative assessment of cell viability and proliferation under the different treatment conditions.

### Cell staining

In a 24-well plate, hBMSCs were initially plated at a density of 50,000 cells per well. On the first day, the cells were maintained in complete medium to facilitate proper adhesion. Beginning on day two, the complete medium was replaced with an osteogenic induction medium, which was refreshed every two days for durations of either 7 or 14 days, depending on the experimental setup. Osteogenic induction medium was composed of growth medium supplemented with 100 nM dexamethasone, 10 mM β-glycerophosphate, and 50 mM ascorbic acid (Sigma–Aldrich, St Louis, MO, USA). After the induction period, the cells underwent three successive washes with PBS to remove any residual medium, followed by fixation in neutral formalin at room temperature for 30 min. Finally, the cells were stained with ALP and ARS solutions as per the standard protocol, and images were subsequently captured using a stereomicroscope.

### Free radical scavenging test

A DPPH• solution at a concentration of 125 µmol/mL was prepared and then mixed in equal volumes with different concentrations of naringenin. The mixture was subsequently incubated at 37 °C for 15 min to allow the reaction to proceed. After the incubation period, the absorbance was measured at 517 nm using a UV-vis spectrophotometer, providing a quantitative assessment of the radical scavenging activity of the naringenin.

To evaluate the scavenging activity of naringenin against hydrogen peroxide, a commercial H₂O₂ detection kit was employed. In this assay, H₂O₂ reacts with ammonium molybdate to generate a stable yellow complex that displays a characteristic absorbance peak at 405 nm. Various concentrations of naringenin were mixed with a 2 mM H₂O₂ solution and incubated at 37 °C for 24 h. After incubation, the residual H₂O₂ was quantified according to the kit protocol provided by the manufacturer. The extent to which naringenin removed H₂O₂ was then determined by comparing the measured concentration with the initial 2 mM level.

Superoxide anions (O₂^•−^) were generated via the enzymatic oxidation of xanthine by xanthine oxidase. These radicals subsequently interacted with water-soluble tetrazolium salt-1 (WST-1) to produce WST-1 formazan, which exhibits a distinct absorption peak at 450 nm. The concentration of the formed formazan was then quantified using a microplate reader set to measure absorbance at 450 nm.

A solution containing 1 mM FeSO₄ and 2 mM H₂O₂ was prepared in sodium acetate buffer, after which various concentrations of naringenin were introduced. The reaction was allowed to proceed for 5 min before the absorbance at 650 nm was measured using TMB (3,3’,5,5’-tetramethylbenzidine) as the chromogenic substrate. In addition, the hydroxyl radical (•OH) scavenging activity of naringenin was further confirmed by electron spin resonance (ESR) spectroscopy. In this complementary assay, •OH radicals produced from the FeSO₄/H₂O₂ reaction in sodium acetate buffer were captured using DMPO (5,5-dimethyl-1-pyrroline N-oxide). Following the addition of different concentrations of naringenin to the reaction system, the reduction in the ESR peak intensity was analyzed to quantify the scavenging effect.

### Antioxidant capacity test

hBMSCs were initially seeded in 24-well plates and divided into four distinct groups: a negative control group, a positive control group treated with 50 mg/mL Rosup, a group exposed solely to 500 µM H₂O₂, and a group receiving a combined treatment of 500 µM H₂O₂ with 50 µM naringenin. Following a 48-hour incubation period, the culture medium was aspirated, and the cells were incubated with a ROS detection solution at 37 °C for 30 min. Subsequent fluorescence imaging was performed using a fluorescence microscope, and the intensity of the fluorescent signals was semi-quantitatively analyzed using ImageJ software.

To determine the total antioxidant capacity imparted by naringenin, hBMSCs were first exposed to 50 µM naringenin for 48 h. After treatment, the cells were harvested and resuspended in 200 µL of cold PBS. The cell suspension was then thoroughly homogenized to achieve complete lysis and to liberate the intracellular antioxidants. The lysate was centrifuged at 12,000 g for 5 min at 4 °C, and the supernatant was collected for further analysis. A standard curve was generated using a Trolox standard solution, after which 170 µL of the assay’s working solution was added to each sample. The mixture was incubated at room temperature for 6 min, and the absorbance was recorded at 414 nm. The total antioxidant capacity was then calculated based on the absorbance values and the established calibration curve, following the manufacturer’s protocol.

For the assessment of superoxide dismutase (SOD) activity, hBMSCs were processed in a similar manner. The cells were first washed three times with PBS to remove any residual media and then lysed using an SOD sample preparation solution. After the addition of the assay’s working reagent, the samples were incubated at 37 °C for 30 min. The absorbance was subsequently measured at 450 nm, and the SOD activity was calculated and expressed in enzyme activity units in accordance with the kit instructions.

In addition, the catalase (CAT) activity was measured by mixing 0.1 mL of the cell lysate supernatant with 1.1 mL of the working solution. The reaction mixture was incubated at 37 °C for 1 min, and then a stop solution was added to terminate the reaction. The final absorbance was measured at 405 nm, and the CAT enzyme activity was calculated based on the absorbance change and the provided protocol.

### Quantitative real-time PCR

Total RNA was extracted from cells or tissues using TRIzol reagent (Invitrogen) in accordance with the manufacturer’s protocol. The isolated RNA was then reverse transcribed into cDNA using a qScript cDNA Synthesis kit (Takara, Shiga, Japan). Quantitative PCR was conducted on an ABI Step-One Plus™ Real-Time PCR System with SYBR^®^ Premix Ex TaqTM (Takara, Shiga, Japan). The expression levels of the target genes were normalized to Gapdh, and the relative quantification was performed using the 2^−ΔΔCt^ method. Table 1 contained a list of all primers.

**Table 1:** Primers for qRT-PCR.

Gene	Forward	Reverse
GAPDH	GGAGCGAGATCCCTCCAAAAT	GGCTGTTGTCATACTTCTCATGG
RUNX2	TGGTTACTGTCATGGCGGGTA	TCTCAGATCGTTGAACCTTGCTA
OPN	GAAGTTTCGCAGACCTGACAT	GTATGCACCATTCAACTCCTCG
OCN	CACTCCTCGCCCTATTGGC	CCCTCCTGCTTGGACACAAAG
ALP	ACTGGTACTCAGACAACGAGAT	ACGTCAATGTCCCTGATGTTATG
iNOS	GGACTTTTGTACTCATCTGCAC	GTGGACGGGTCGATGTCAC
TNF-α	GGCCAATGTGAGGGAGTTGAT	CCCGCTTTATCTGTGAGCCC
COX-2	GCACCCCGACATAGAGAGC	CTGCGGAGTGCAGTGTTCT
IL-6	CACTGGTCTTTTGGAGTTTGAG	GGACTTTTGTACTCATCTGCAC
AKR1B1	TTTTCCCATTGGATGAGTCGG	CCTGGAGATGGTTGAAGTTGG
SNCA	AAGAGGGTGTTCTCTATGTAGGC	GCTCCTCCAACATTTGTCACTT

### Gene expression data collection and preprocessing

The GSE176086 dataset was obtained from the Gene Expression Omnibus (GEO) database (http://www.ncbi.nlm.nih.gov/geo) and is based on the GPL21185 platform. This dataset comprises 12 hBMSC samples, including six samples treated with TNF-α and six untreated controls. The online analysis tool GEO2R (https://www.ncbi.nlm.nih.gov/geo/geo2r) was employed for data normalization and differential gene expression (DEG) analysis. The selection criteria for DEGs were set as an absolute log2 fold change (logFC) greater than 1 and a P-value less than 0.05. Furthermore, GEO2R was also utilized to generate visualizations such as volcano plots to provide an intuitive representation of gene expression significance and variation magnitude.

### Functional analysis

The Metascape database (http://metascape.org) was utilized to conduct Gene Ontology (GO) enrichment analyses, encompassing biological processes, cellular components, and molecular functions, for the previously identified differentially expressed genes (DEGs). Additionally, Kyoto Encyclopedia of Genes and Genomes (KEGG) pathway analyses were performed to further elucidate the functional roles of these DEGs. A significance threshold of *p* < 0.05 was applied for all analyses. The KEGG pathway data used in this study was obtained with permission from Kanehisa Laboratories^23,24^.

### Gene set enrichment analysis (GSEA)

GSEA was performed using the GOBP_OSSIFICATION and CYTOKINE_CYTOKINE_RECEPTOR_INTERACTION gene sets obtained from the Molecular Signatures Database (MSigDB). The DEGs identified in the GSE176086 dataset were uploaded to the Wei Sheng Xin online platform (https://www.bioinformatics.com.cn/) to conduct GSEA, enabling systematic evaluation of pathway enrichment patterns associated with osteogenic suppression and inflammatory signaling.

### Protein-protein interaction (PPI) network analysis

We used STRING 11.0 (https://string-db.org/), an online tool for analyzing protein interactions, to identify the network PPI. All DEGs were uploaded for PPI analysis. To further capture the relationships between the terms, a subset of enriched terms has been selected and rendered as a network plot, where terms with a similarity > 0.3 are connected by edges. We select the terms with the best p-values from each of the 20 clusters, with the constraint that there are no more than 15 terms per cluster and no more than 250 terms in total. The three most significantly enriched clusters were selected for further refined PPI network analysis. Cytoscape v3.9.1 (https://www.cytoscape.org/), a tool for analyzing and visualizing protein interaction networks, was used to display the nodes and connections in the PPI network.

### Naringenin target prediction

We utilized SwissTargetPrediction (http://www.swisstargetprediction.ch/) to identify potential target proteins of naringenin and conducted enrichment analysis on all predicted targets. To refine our results, we intersected the top 100 predicted target genes with the top 200 DEGs from the GSE176086 dataset. This intersection analysis revealed two potential target genes, AKR1B1 and SNCA, which may play a critical role in the mechanism of naringenin’s action.

### Molecular Docking

To predict the binding interactions between naringenin and its potential target proteins AKR1B1 and SNCA, molecular docking analysis was performed using the CB-Dock2 online tool (http://clab.labshare.cn/cb-dock2/). The three-dimensional (3D) structure of naringenin was retrieved from the PubChem database, while the protein structures of AKR1B1 and SNCA were obtained from the Protein Data Bank (PDB). Before docking, the protein structures were prepared by removing water molecules and heteroatoms, followed by energy minimization. CB-Dock2 employs blind docking to automatically predict the binding pocket and perform flexible docking simulations. The docking scores, which represent the binding affinity, were recorded, and the docking conformations with the lowest binding energy were selected for further analysis.

### Molecular dynamic simulation

Molecular dynamics (MD) simulations were conducted on the WebGro online platform with the GROMOS96 43a1 force field and the TIP4P water model. The system was solvated in a dodecahedron box, and 0.15 M NaCl ions were added to mimic physiological ionic strength. Long-range electrostatic interactions were calculated using the Particle Mesh Ewald method with a cutoff distance of 1.2 nm, while short-range van der Waals interactions were truncated at 1.0 nm. Bond lengths involving hydrogen atoms were constrained using the LINCS algorithm, enabling a 2 fs integration time step.

The system underwent energy minimization using the steepest descent algorithm to eliminate steric clashes, followed by a two-step equilibration protocol: (1) 100 ps of NVT (constant particle number, volume, and temperature) equilibration with the V-rescale thermostat to stabilize the temperature at 298 K, and (2) 100 ps of NPT (constant particle number, pressure, and temperature) equilibration using the Berendsen barostat to adjust the pressure to 1 bar. Subsequently, a production MD simulation was carried out for 50 ns under NPT conditions, with coordinates saved every 10 ps for trajectory analysis. Trajectory analysis included root mean square deviation (RMSD) and root mean square fluctuation (RMSF).

### Statistical analysis

All statistical analyses were performed using GraphPad Prism (version 8.2.0, GraphPad Software, CA, USA). Data from each experiment were expressed as means ± standard deviation (SD), and all experiments were conducted in triplicate or more. Prior to comparisons, all datasets met the assumptions of normality (assessed by the Shapiro-Wilk test) and homogeneity of variances (assessed by Levene’s test). For comparisons between two groups, the Student’s t-test was used. For comparisons involving multiple groups, One-way analysis of variance (ANOVA) followed by Dunnett’s multiple comparisons test was employed. Statistical significance was considered for p-values < 0.05.

## Results

### Naringenin ameliorates TNF-α-induced suppression of osteogenic differentiation in hBMSCs

**Fig. 1:**
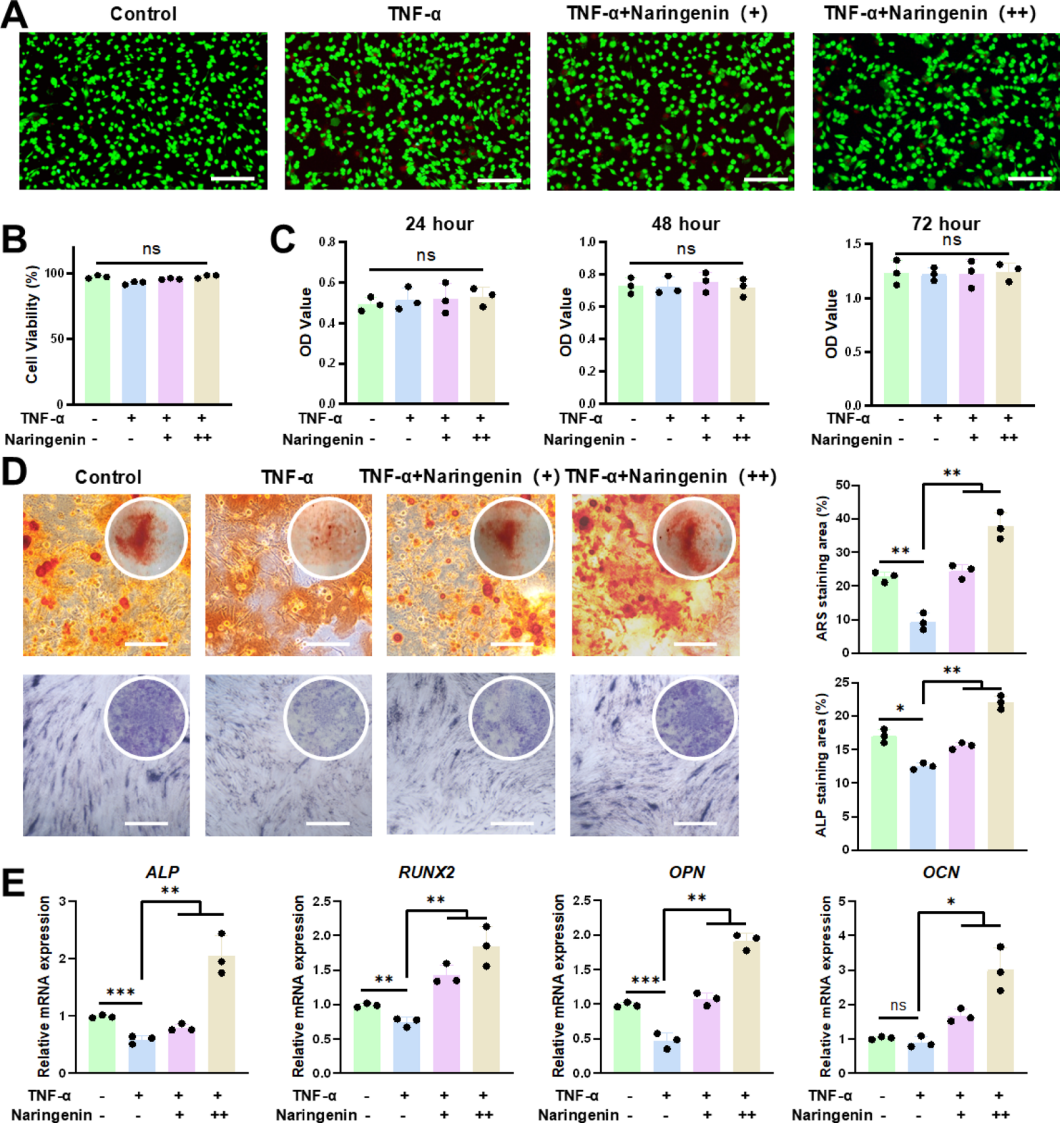
Naringenin ameliorates TNF-α-induced suppression of osteogenic differentiation in hBMSCs. (**A**,**B**) Live/Dead images of hBMSCs cultured for 48 h. Scale bar: 200 μm. (**C**) Viability of hBMSCs quantified by the CCK-8 assay. (**D**) ARS and ALP staining of hBMSCs cultured for 14 days and 7 days respectively, along with their semi-quantitative analysis. Scale bar: 100 μm. (**E**) qRT-PCR analysis of osteogenic markers, including RUNX2, OPN, OCN and ALP. All data are shown as the means ± SD (*n* = 3) (∗*p* < 0.05, ∗∗*p* < 0.01, ∗∗∗*p* < 0.001).

To evaluate the potential of naringenin in reversing TNF-α-induced inhibition of osteogenic differentiation, we first assessed its effects on the survival and proliferation of hBMSCs. Live/Dead staining (Fig. 1A-B) and CCK-8 assays (Fig. 1C) revealed that TNF-α did not significantly affect the cell viability or proliferation at the tested concentrations, and similarly, both low and high doses of naringenin did not show any adverse effects on cell growth.

Next, we explored whether naringenin could rescue osteogenic differentiation that was suppressed by TNF-α. Both ALP and ARS staining demonstrated that TNF-α significantly inhibited osteogenic differentiation, whereas naringenin effectively reversed this inhibition in a dose-dependent manner (Fig. 1D). Additionally, qRT-PCR analysis confirmed that the addition of naringenin led to a marked upregulation of osteogenic markers (Fig. 1E). These findings collectively suggest that naringenin can counteract the inhibitory effects of TNF-α on osteogenesis, supporting its potential as a therapeutic agent for conditions involving osteogenic dysfunction.

### Anti-inflammatory and antioxidant properties of naringenin

Having established that naringenin can reverse TNF-α-induced inhibition of osteogenesis, we proceeded to investigate the underlying molecular mechanisms through which naringenin exerts its beneficial effects on osteogenic differentiation. Given that TNF-α is a well-known inflammatory mediator involved in various pathophysiological processes, we aimed to explore whether naringenin also possesses anti-inflammatory and antioxidant properties that could further support its role in modulating osteogenesis.

**Fig. 2:**
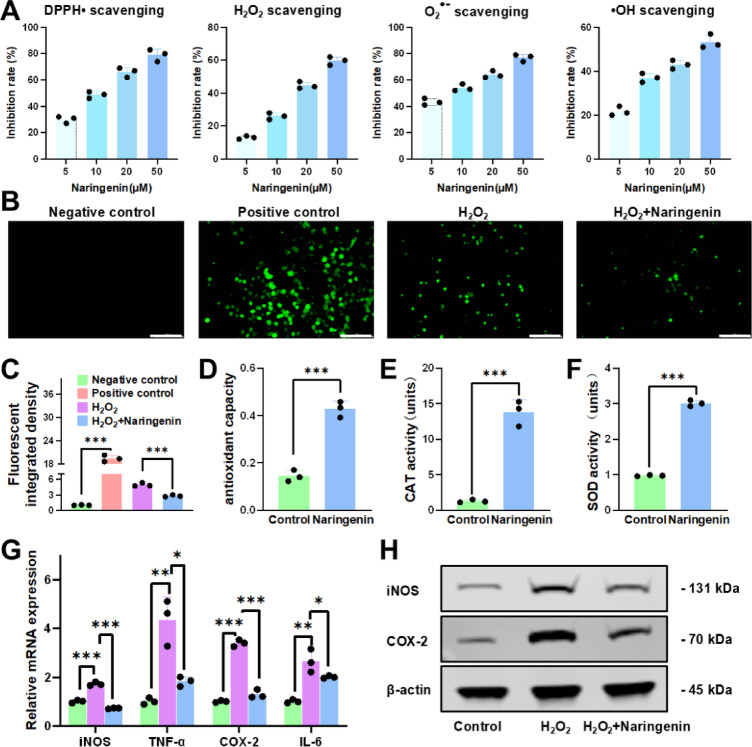
Anti-inflammatory and antioxidant properties of naringenin. (**A**) Scavenging effect of naringenin with different concentration on free radicals. (**B**,**C**) Fluorescence detection of ROS. Scale bar: 100 μm. (**D**) Total antioxidant capacity of naringenin. (**E**) CAT activity of naringenin. (**F**) SOD activity of naringenin. (**G**,**H**) Gene and protein expression analysis of inflammatory markers. All data are shown as the means ± SD (*n* = 3) (∗*p* < 0.05, ∗∗*p* < 0.01, ∗∗∗*p* < 0.001).

To assess the antioxidant capacity of naringenin, we initially evaluated the ability of naringenin to scavenge various free radicals, including DPPH•, •OH, O_2_^•−^ and H_2_O_2_ (Fig. 2A). The results demonstrated that naringenin effectively neutralizes these free radicals in a dose-dependent manner. Then we measured the intracellular ROS levels using fluorescence detection (Fig. 2B). The results revealed a significant reduction in ROS content following naringenin treatment, indicating its potential to alleviate oxidative stress (Fig. 2C). To further evaluate its antioxidant potential, we performed three distinct assays: total antioxidant capacity, SOD activity, and CAT activity (Fig. 2D-F). The findings consistently demonstrated the robust antioxidant effects of naringenin.

Moreover, we investigated the impact of naringenin on inflammatory cytokine expression under oxidative stress conditions induced by H_2_O_2_. Exposure to H_2_O_2_ led to a marked upregulation of several inflammatory mediators, including TNF-α, iNOS, COX-2, and IL-6 (Fig. 2G). However, naringenin treatment significantly attenuated the expression of these inflammatory cytokines, indicating that naringenin possesses potent anti-inflammatory properties. These results suggest that naringenin not only mitigates oxidative stress but also effectively modulates inflammation, highlighting its potential as a therapeutic agent for inflammatory-related bone disorders.

### TNF-α-induced transcriptional reprogramming of hBMSCs impairs osteogenesis and enhances inflammatory pathways

**Fig. 3:**
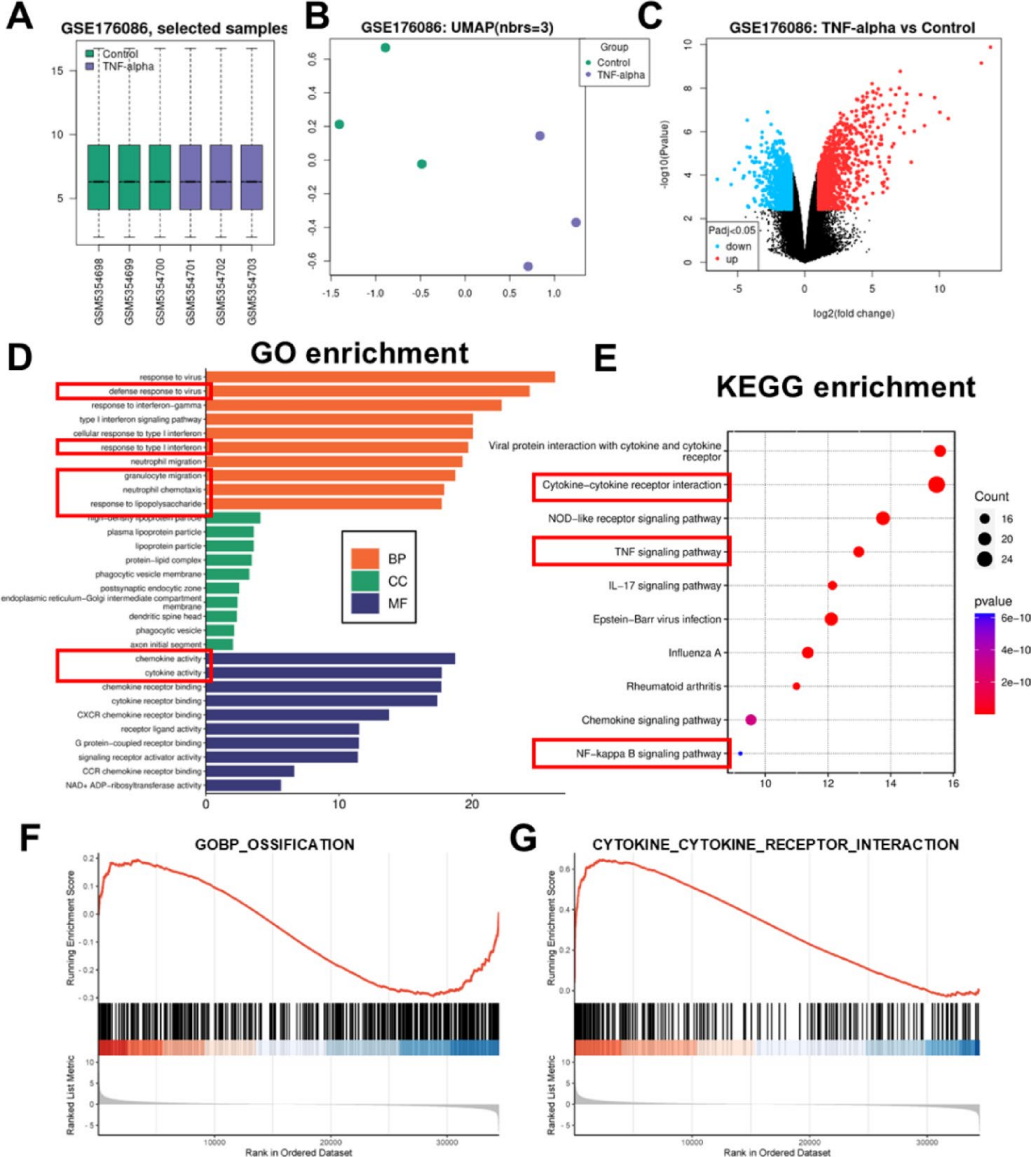
TNF-α-induced transcriptional reprogramming of hBMSCs impairs osteogenesis and enhances inflammatory pathways. (**A**) Standardization of GSE176086 datasets. (**B**) UMAP of GSE176086 datasets. (**C**) Volcano plot of the DEGs in GSE176086. (**D**) GO functional annotation (BP, CC, and MF) for the DEGs. (**E**) KEGG pathway enrichment analysis for the DEGs. The KEGG pathway data used in this study was obtained with permission from Kanehisa Laboratories^23,24^. (**F**,**G**) Gene set enrichment analysis (GSEA) analysis.

Next, through GEO database, we found and downloaded the mRNA microarray analysis dataset GSE176086 after TNF-α treatment of hBMSCs. After normalizing the data, differential expression analysis revealed 1,095 significantly upregulated genes and 1,083 downregulated genes, which were visualized in a volcano plot (Fig. 3A-C). GO enrichment analysis of the top three biological processes indicated significant associations with viral response (e.g., defense response to virus, type I interferon signaling), chemotaxis (e.g., granulocyte migration), and inflammatory activation (e.g., response to lipopolysaccharide), reflecting TNF-α’s role in triggering immune-inflammatory cascades (Fig. 3D). KEGG pathway analysis further confirmed enrichment in pathways related to cytokine-cytokine receptor interaction, NF-κB signaling, and TNF signaling (Fig. 3E), underscoring TNF-α’s dual role in suppressing osteogenesis and enhancing pro-inflammatory signaling.

To explore the mechanistic relationship between TNF-α and osteogenic inhibition, GSEA was conducted using the GOBP_OSSIFICATION and CYTOKINE_CYTOKINE_RECEPTOR_INTERACTION gene sets. The results showed that TNF-α treatment negatively correlated with GOBP_OSSIFICATION (NES = -2.1, FDR < 0.05; Fig. 3F), indicating a suppression of osteogenic differentiation. Conversely, CYTOKINE_CYTOKINE_RECEPTOR_INTERACTION exhibited significant positive enrichment (NES = 2.6, FDR < 0.05; Fig. 3G), consistent with TNF-α-induced inflammatory activation.

**Fig. 4:**
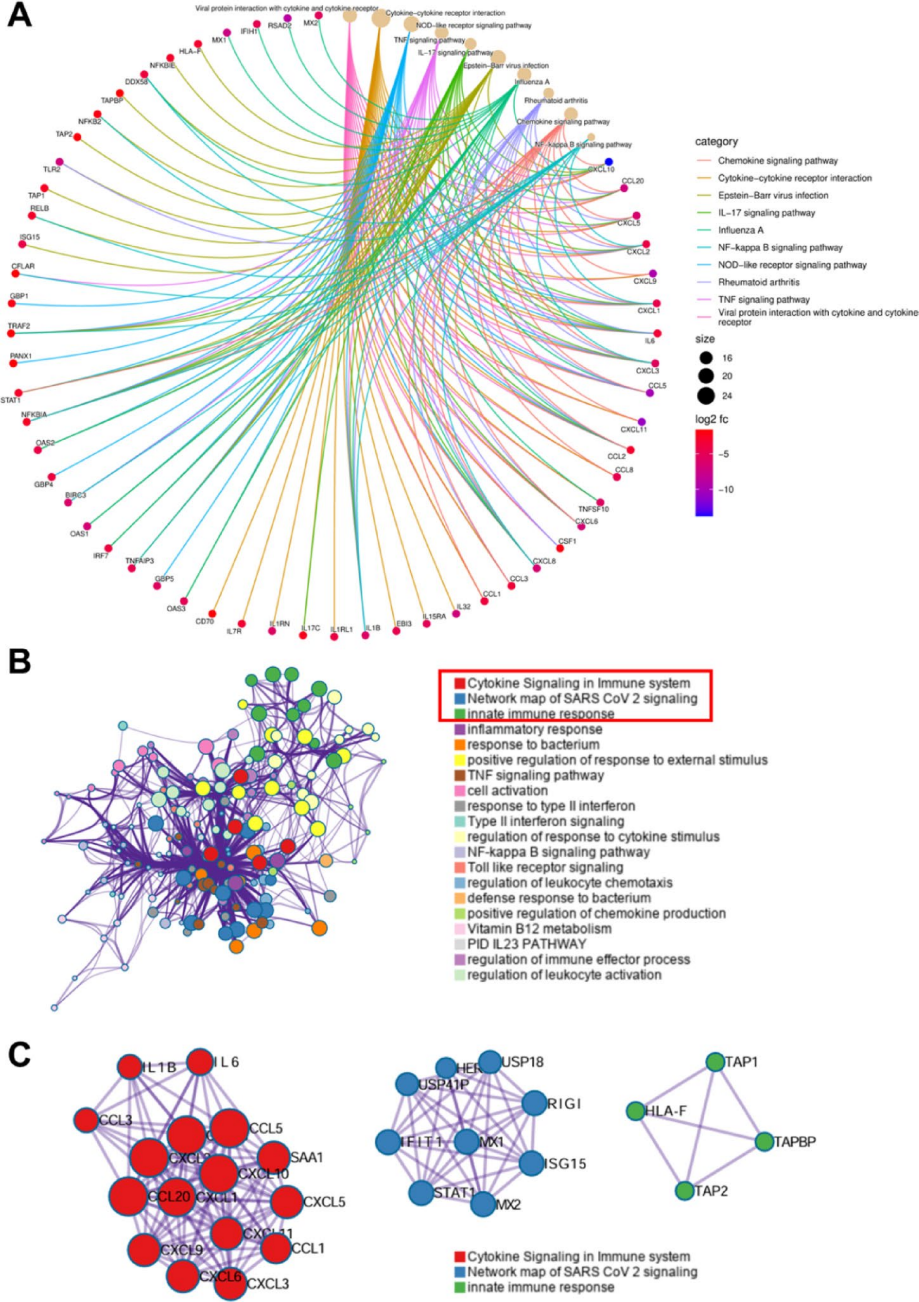
The Protein-Protein Interaction network. (**A**) Enrichment analysis string diagram of difference categories and high significance DEGs. (**B**) TOP 20 enriched biological categories. (**C**) PPI networks of the TOP 3 enriched biological categories.

Furthermore, we examined the association between the top ten enriched pathways and representative DEGs with significant expression changes (Fig. 4A). Concurrently, a PPI network was constructed using an online database, followed by functional enrichment analysis of the identified DEGs. The results indicated that these DEGs were primarily enriched in 20 biological categories (Fig. 4B). To refine our analysis, we selected the top three most significantly enriched categories for further PPI network investigation. The findings revealed that these categories were highly correlated with key DEGs, predominantly associated with cytokine signaling in the immune system, the network map of SARS-CoV-2 signaling, and the innate immune response (Fig. 4C). Collectively, these findings demonstrate that TNF-α drives a transcriptional reprogramming of BMSCs, shifting them towards an inflammatory phenotype while impairing their osteogenic potential, providing a molecular framework for its detrimental effects on bone formation.

### Naringenin reverses TNF-α-induced osteogenic Inhibition by regulating AKR1B1

**Fig. 5:**
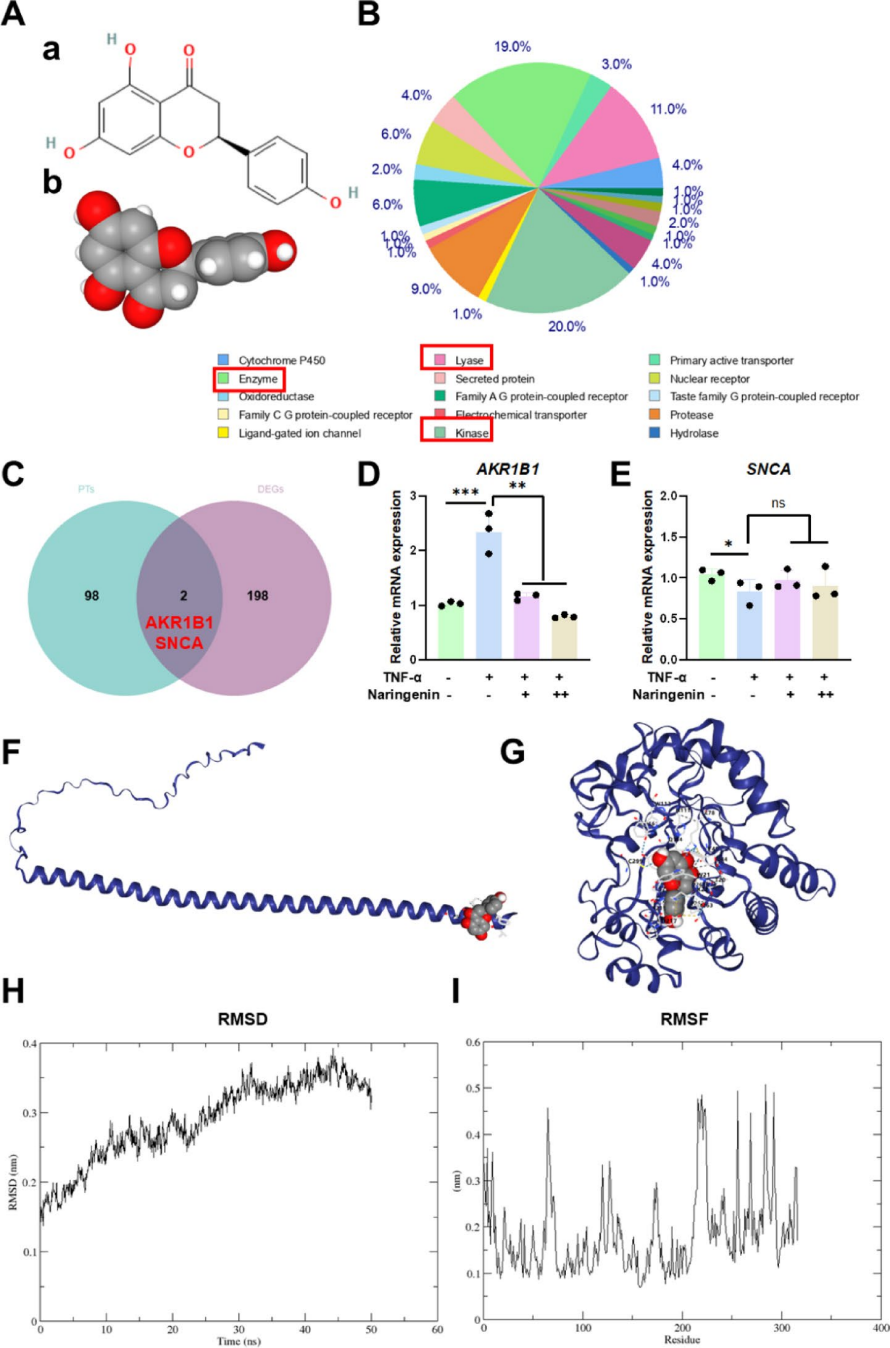
Naringenin’s target prediction and molecular structure analysis. (**A**) The chemical structure (a) and molecular structure (b) of naringenin. (**B**) Target prediction and enrichment analysis of naringenin. (**C**) Wayne diagram between DEGs and the predicted targets. (**D**,**E**) qRT-PCR analysis of AKR1B1 and SNCA. (**F**,**G**) Molecular docking simulations of naringenin-AKR1B1 and naringenin-SNCA. (**H**,**I**) RMSD and RMSF analysis of naringenin-AKR1B1 complex. All data are shown as the means ± SD (*n* = 3) (∗*p* < 0.05, ∗∗*p* < 0.01, ∗∗∗*p* < 0.001).

To further elucidate the molecular mechanism underlying naringenin’s protective effects, we first predicted its potential targets (PTs) based on its chemical structure and performed enrichment analysis on the identified targets (Fig. 5A-B). The results revealed that the PTs were primarily enriched in kinase, enzyme, and lyase categories, suggesting that naringenin predominantly interacts with enzymatic proteins.

Next, we intersected the top 100 PTs with the top 200 DEGs identified in the GSE176086 dataset, leading to the identification of two potential key genes: AKR1B1 and SNCA (Fig. 5C). quantitative RT-PCR analysis demonstrated that TNF-α treatment significantly upregulated AKR1B1 expression, whereas the addition of naringenin effectively suppressed this effect (Fig. 5D). In contrast, SNCA expression did not exhibit significant changes upon treatment (Fig. 5E).

To gain further insights into the interaction between naringenin and these candidate genes, molecular docking simulations were conducted. The docking results indicated that naringenin exhibited a strong binding affinity for AKR1B1 (Vina score = -10), whereas its binding affinity for SNCA was relatively weaker (Vina score = -5) (Fig. 5F-G). Furthermore, the naringenin-AKR1B1 complex was selected for molecular dynamics simulations. RMSD and RMSF served as critical metrics to evaluate system stability, where values within 0.5 nm were considered indicative of a stable system. Throughout the simulation, the naringenin-AKR1B1 complex exhibited favorable stability, with both RMSD and RMSF consistently maintained below the threshold of 0.5 nm, reflecting robust structural integrity and minimal conformational fluctuations under the defined simulation conditions (Fig. 5H-I). These findings strongly suggest that naringenin may exert its biological effects by directly binding to AKR1B1.

Functionally, AKR1B1 encodes aldose reductase, an enzyme that catalyzes the conversion of glucose to sorbitol, consuming NADPH in the process^25^. This diminishes glutathione (GSH) regeneration capacity and exacerbates oxidative stress. Our results suggest that naringenin may attenuate the activation of the polyol pathway by directly binding to the active site of AKR1B1, thereby preserving NADPH for GSH regeneration and reducing ROS levels. Additionally, naringenin may inhibit AKR1B1 expression through its antioxidant (ROS-reducing) and anti-inflammatory (NF-κB nuclear translocation-inhibiting) properties.

**Fig. 6:**
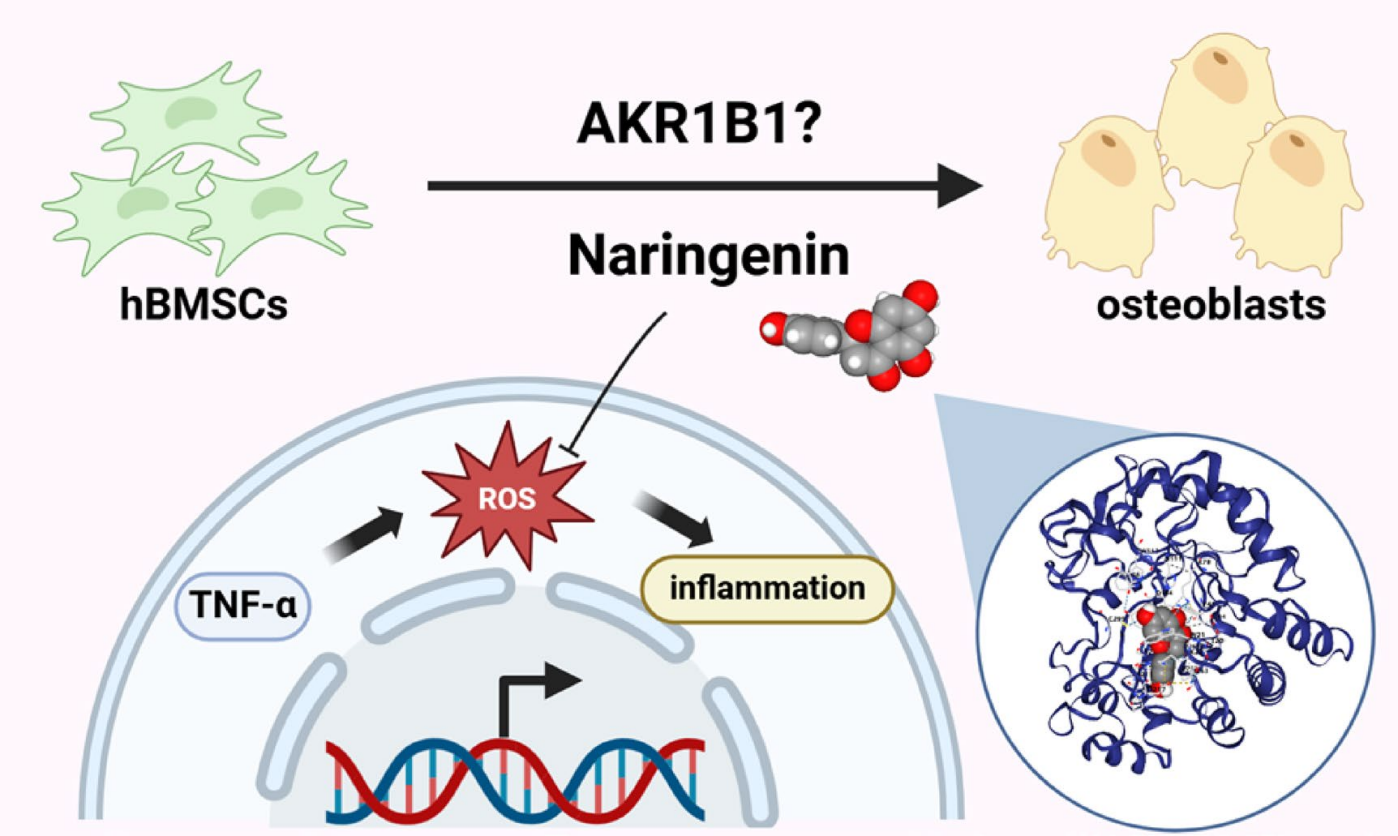
Naringenin exerts antioxidant and anti-inflammatory effects by regulating AKR1B1 and reverses the osteogenic inhibition induced by TNF-α.

Collectively, our findings provide strong evidence that naringenin reverses TNF-α-induced osteogenic inhibition and may function by regulating AKR1B1, highlighting it as a therapeutic agent against inflammation-induced bone loss (Fig. 6).

## Discussion

Chronic inflammatory microenvironments, driven by cytokines such as TNF-α, disrupt bone remodeling by skewing the balance toward osteoclastogenesis and suppressing osteoblast differentiation, ultimately leading to pathological bone loss^26,27^. This study elucidates the therapeutic potential of naringenin, a citrus-derived flavonoid, in counteracting TNF-α-induced osteogenic suppression in hBMSCs. Our findings highlight a novel mechanism by which naringenin rescues osteoblast differentiation through direct interaction with AKR1B1, a key enzyme implicated in oxidative stress and inflammation. These results not only advance our understanding of flavonoid-based interventions for inflammatory bone disorders but also underscore the translational potential of targeting AKR1B1 in metabolic and inflammatory bone diseases.

The clinical relevance of this work lies in the urgent need for therapies that address the dual challenges of inflammation and impaired osteogenesis in conditions such as rheumatoid arthritis, diabetic osteopathy, and osteoporosis^28,29^. For instance, osteomyelitis, a devastating bone infection, represents a quintessential model of inflammatory bone destruction^30^. The persistent bacterial presence triggers an overwhelming and dysregulated immune response, characterized by the infiltration of neutrophils and macrophages and the robust production of cytokines, including high levels of TNF-α, IL-1β, and IL-6^31–33^. This cytokine storm not only facilitates bone resorption by activating osteoclasts but also directly suppresses the osteogenic differentiation of BMSCs, leading to progressive bone necrosis and impaired healing—a pathophysiology that aligns precisely with the mechanistic insights of our study^34,35^. While TNF-α inhibitors like biologics alleviate inflammation, their immunosuppressive risks and inability to directly restore osteoblast function limit their utility^36^. Natural compounds like naringenin, with dual anti-inflammatory and osteoprotective properties, offer a promising alternative. Our study demonstrates that naringenin reverses TNF-α-driven suppression of osteogenic markers (e.g., RUNX2, OPN, ALP) while mitigating oxidative stress and inflammatory cytokine production. This multifaceted action positions naringenin as a therapeutic candidate capable of addressing both the inflammatory and metabolic dysregulation underlying bone loss.

Central to this mechanism is the identification of AKR1B1 as a critical mediator of TNF-α’s detrimental effects. AKR1B1, encoding aldose reductase, catalyzes the polyol pathway, consuming NADPH to convert glucose into sorbitol^25,37^. This process depletes cellular NADPH reserves, impairing GSH regeneration and exacerbating ROS accumulation. Elevated ROS levels activate NF-κB, further amplifying inflammatory cascades and suppressing osteogenic transcription factors^38–40^. Our data reveal that TNF-α significantly upregulates AKR1B1 expression in hBMSCs, aligning with prior studies linking AKR1B1 to diabetic complications and oxidative tissue damage. Crucially, naringenin suppresses AKR1B1 at both transcriptional and functional levels. Molecular docking analysis revealed a strong binding affinity (Vina score = -10) between naringenin and AKR1B1’s active site, suggesting direct inhibition of its enzymatic activity. By preserving NADPH for GSH synthesis, naringenin reduces oxidative burden, thereby restoring redox homeostasis—a finding corroborated by its dose-dependent scavenging of free radicals (DPPH•, •OH, O₂•⁻) and enhancement of SOD/CAT activity.

The rescue of osteogenesis by naringenin cannot be attributed solely to AKR1B1 inhibition. Its anti-inflammatory effects, particularly the suppression of TNF-α-induced NF-κB activation, play a complementary role. TNF-α triggers NF-κB nuclear translocation, upregulating pro-inflammatory cytokines (e.g., IL-6, COX-2) that further inhibit osteoblast differentiation^41–43^. By reducing ROS levels, naringenin disrupts ROS-dependent NF-κB activation, while its potential interaction with IKK or IκBα may block downstream signaling^44,45^. This dual inhibition of oxidative and inflammatory pathways creates a permissive microenvironment for osteogenic differentiation, as evidenced by the restoration of RUNX2 and Osterix expression in naringenin-treated hBMSCs. Notably, our bioinformatics approach intersecting naringenin’s predicted targets with TNF-α-regulated DEGs initially identified SNCA as a potential candidate. However, experimental validation revealed no significant changes in SNCA expression, suggesting that naringenin’s osteoprotective effects are predominantly mediated through AKR1B1. This discrepancy may arise from tissue-specific regulatory mechanisms or post-transcriptional modifications not captured in our assays, warranting further investigation into SNCA’s role in BMSC differentiation.

The pivotal role of AKR1B1 in naringenin’s mechanism highlights its potential as a therapeutic target for inflammatory bone diseases. AKR1B1 inhibitors, such as epalrestat, are already clinically approved for diabetic neuropathy or lung cancer, underscoring the enzyme’s druggability^46–48^. Our work extends this paradigm to bone metabolism, proposing that AKR1B1 inhibition could mitigate inflammation-driven osteopenia. However, several unanswered questions remain. While molecular docking supports naringenin-AKR1B1 binding, structural validation (e.g., X-ray crystallography) is needed to confirm interaction details. Specifically, future work should determine if AKR1B1 knockout abrogates TNF-α’s inhibitory effects on osteogenesis and whether constitutive AKR1B1 expression can resist the restorative effects of naringenin. These experiments are essential and will be a primary focus of our subsequent research. Additionally, the contribution of AKR1B1-independent pathways, such as naringenin’s modulation of BMP/Smad or MAPK signaling, requires exploration. Furthermore, our study focused on in vitro models, which may not fully recapitulate the complexity of bone tissue microenvironments. Co-culture systems or 3D bone organoids could better model interactions between osteoblasts, osteoclasts, and immune cells.

Despite these limitations, our findings provide compelling evidence for naringenin’s osteoprotective effects. However, several gaps must be addressed to advance translational applications. First, the dose-dependent effects of naringenin were only partially characterized. A broader concentration range and pharmacokinetic studies are needed to establish optimal therapeutic doses^49^. It is widely recognized that the oral bioavailability of free naringenin is relatively low, and achieving high micromolar concentrations in systemic circulation in vivo is challenging. This is a common hurdle for many dietary polyphenols. However, in vitro models require higher concentrations to elicit measurable effects due to factors like serum binding and rapid cellular metabolism, which is why the range of 10–100 µM is standardly used in cell culture studies to probe mechanisms of action^50^. The use of nanoparticle-based delivery systems, phospholipid complexes, or other formulation strategies has been shown to significantly enhance the bioavailability and tissue distribution of naringenin^51^. Second, the lack of in vivo data limits clinical relevance. Future work should assess naringenin’s efficacy in murine models of osteoporosis or fracture healing, particularly in the context of chronic inflammation. Third, the role of SNCA, though not validated here, warrants further investigation. Its involvement in protein aggregation and mitochondrial function suggests potential crosstalk with oxidative stress pathways in BMSCs. CRISPR-based knockout or overexpression studies could clarify its contribution to osteogenic differentiation.

In conclusion, this study establishes naringenin as a potent modulator of TNF-α-induced osteogenic suppression, primarily through targeting AKR1B1. By inhibiting AKR1B1, naringenin restores NADPH availability, attenuates oxidative stress, and disrupts NF-κB-driven inflammation, thereby rescuing osteoblast differentiation. These findings bridge the gap between phytochemical research and clinical orthopedics, offering a mechanistic rationale for repurposing naringenin or AKR1B1 inhibitors in inflammatory bone diseases. Future studies should prioritize in vivo validation and explore combinatorial therapies targeting both oxidative stress and inflammatory pathways. Ultimately, this work advances our understanding of flavonoid-based interventions and underscores the therapeutic potential of dual-action agents in metabolic and inflammatory bone disorders.

## Conclusion

This study establishes naringenin as a potent modulator of TNF-α-induced osteogenic suppression, primarily through targeting AKR1B1. By inhibiting AKR1B1, naringenin restores NADPH availability, attenuates oxidative stress, and disrupts NF-κB-driven inflammation, thereby rescuing osteoblast differentiation. These findings bridge the gap between phytochemical research and clinical orthopedics, offering a mechanistic rationale for repurposing naringenin or AKR1B1 inhibitors in inflammatory bone diseases. Future studies should prioritize in vivo validation and explore combinatorial therapies targeting both oxidative stress and inflammatory pathways. Ultimately, this work advances our understanding of flavonoid-based interventions and underscores the therapeutic potential of dual-action agents in metabolic and inflammatory bone disorders.

## Data Availability

The data that support the findings of this study are available from the corresponding author upon reasonable request.

## Abbreviations

TNF: αTumor Necrosis Factor-alpha
BMSCs: Bone marrow sesenchymal stem cells
ROS: Reactive oxygen species
GO: Gene ontology
KEGG: Kyoto encyclopedia of genes and genomes
PPI: Protein-protein interaction
WST-1: Water-soluble tetrazolium salt-1
SOD: Superoxide dismutase
CAT: Catalase
GEO: Gene expression omnibus
DEGs: Differentially expressed genes
GSEA: Gene set enrichment analysis
PTs: Potential targets
GSH: Glutathione
MD: Molecular dynamics
RMSD: Root mean square deviation
RMSF: Root mean square fluctuation
